# Supplementary material for: Structural polymorphism of α-synuclein fibrils alters the pathway of Hsc70-mediated disaggregation
Source: EMBO J. 2025 Oct 6;44(22):6499–526. doi: 10.1038/s44318-025-00573-3 (PMC12623964; doi:10.1038/s44318-025-00573-3)
Supplement: Supplementary file 1 — Appendix [file 44318_2025_573_MOESM1_ESM.pdf]

Appendix for “Structural polymorphism of α-synuclein fibrils alters pathway of Hsc70 mediated disaggregation”

Table of contents

Appendix Figure S1 …… 2

Appendix Figure S2 …… 3

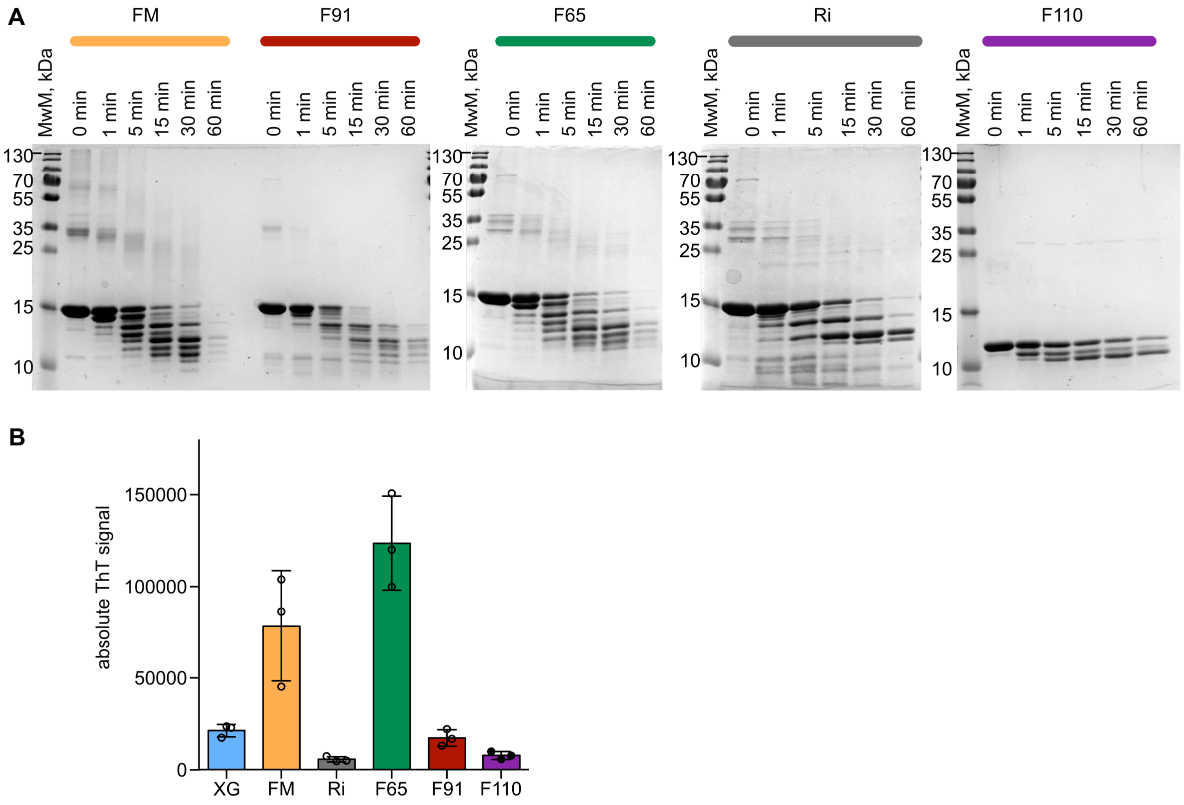

Appendix Figure S1 | α-syn polymorphs differ in sensitivity to proteolysis and reactivity to Thioflavin T.

A SDS-PAGE gels of limited proteolysis of FM (yellow), F91 (red), F65 (green), Ri (grey), and the C-terminally truncated F110 (purple) by proteinase K at timepoint 0, and after 1, 5, 15, 30 and 60 min. **B** Absolute ThT signal of α-syn fibrillar polymorphs (XG (blue), FM (yellow), Ri (grey), F65 (green), F91 (red), and the C-terminally truncated F110 (purple)) at the same concentration (2 µM protein – 30 µM ThT). Data are mean ± s.e.m. of three biological replicates.

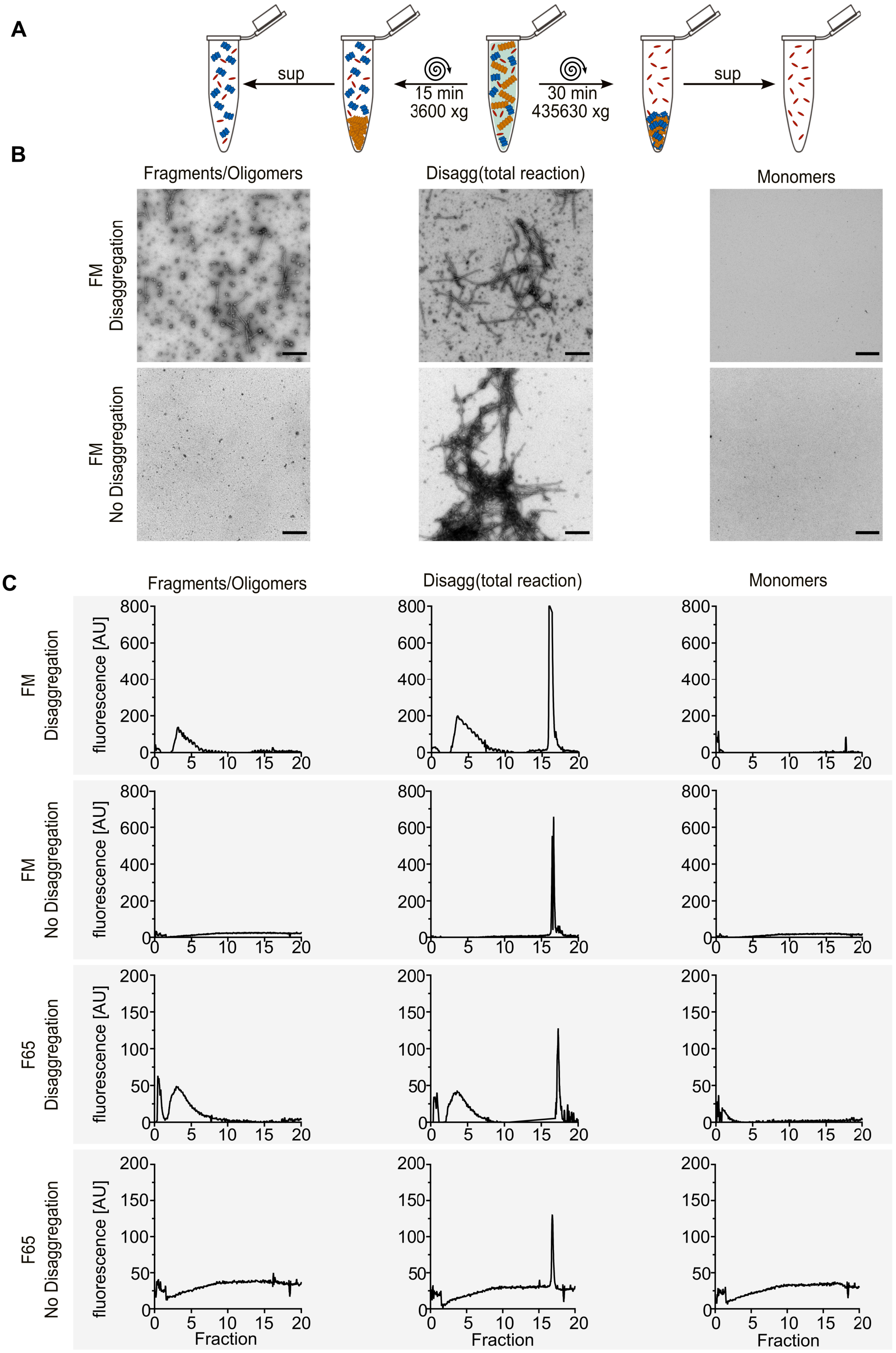

Appendix Figure S2 | Disaggregation reaction products can be fractioned based on differential centrifugation.

A Centrifugation procedure as shown in Fig. 4C to separate different disaggregation reaction products. A total disaggregation reaction is centrifuged at 3,600g for 15 min, fibrils (orange) are separated in the pellet and small fragments/oligomers remain in the supernatant (left). By centrifugation of the total reaction at a higher speed (435,630g) for 30 min, small fragments/oligomers (blue) and fibrils are pelleted and only monomers (red) remain in the supernatant (right). **B** Representative electron micrographs of fibrils incubated with the chaperone machinery in the presence (Disaggregation) and absence (No Disaggregation) of ATP for the polymorph FM. Scale bar 500 nm. **C** Sucrose density gradient (10-85%) profile of AF555-labelled fibrils incubated with the active (Disaggregation) and inactive (No Disaggregation) chaperone machinery and similar prepared as in panel A.
