# Supplementary figures and images for: Structural polymorphism of α-synuclein fibrils alters the pathway of Hsc70-mediated disaggregation
Source: EMBO J. 2025 Oct 6;44(22):6499–526. doi: 10.1038/s44318-025-00573-3 (PMC12623964; doi:10.1038/s44318-025-00573-3)

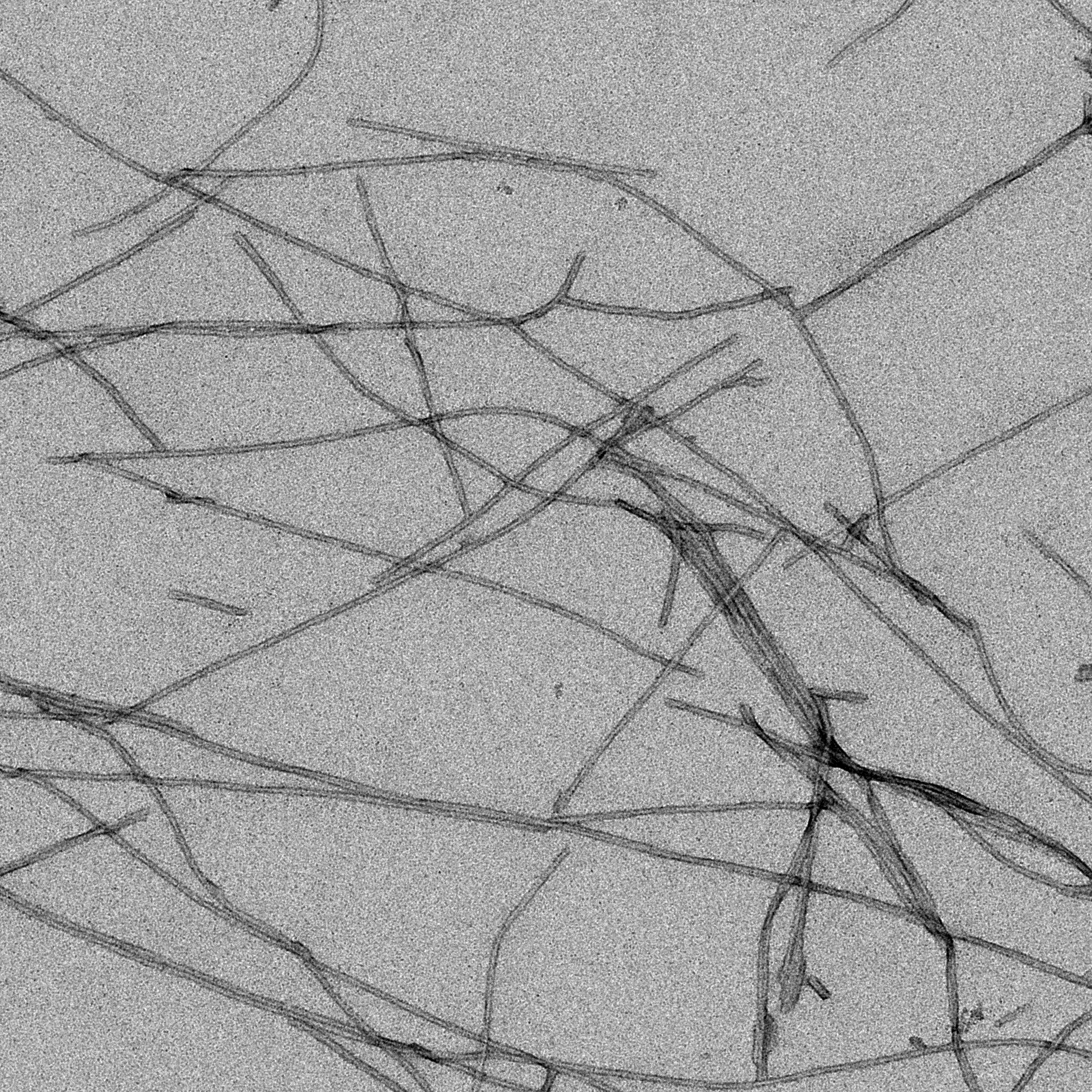

Supplement: Supplementary file 3 — Source data Fig. 1 [file 44318_2025_573_MOESM3_ESM.zip › Figure 1/1A/F65_10kX_0036.jpg]

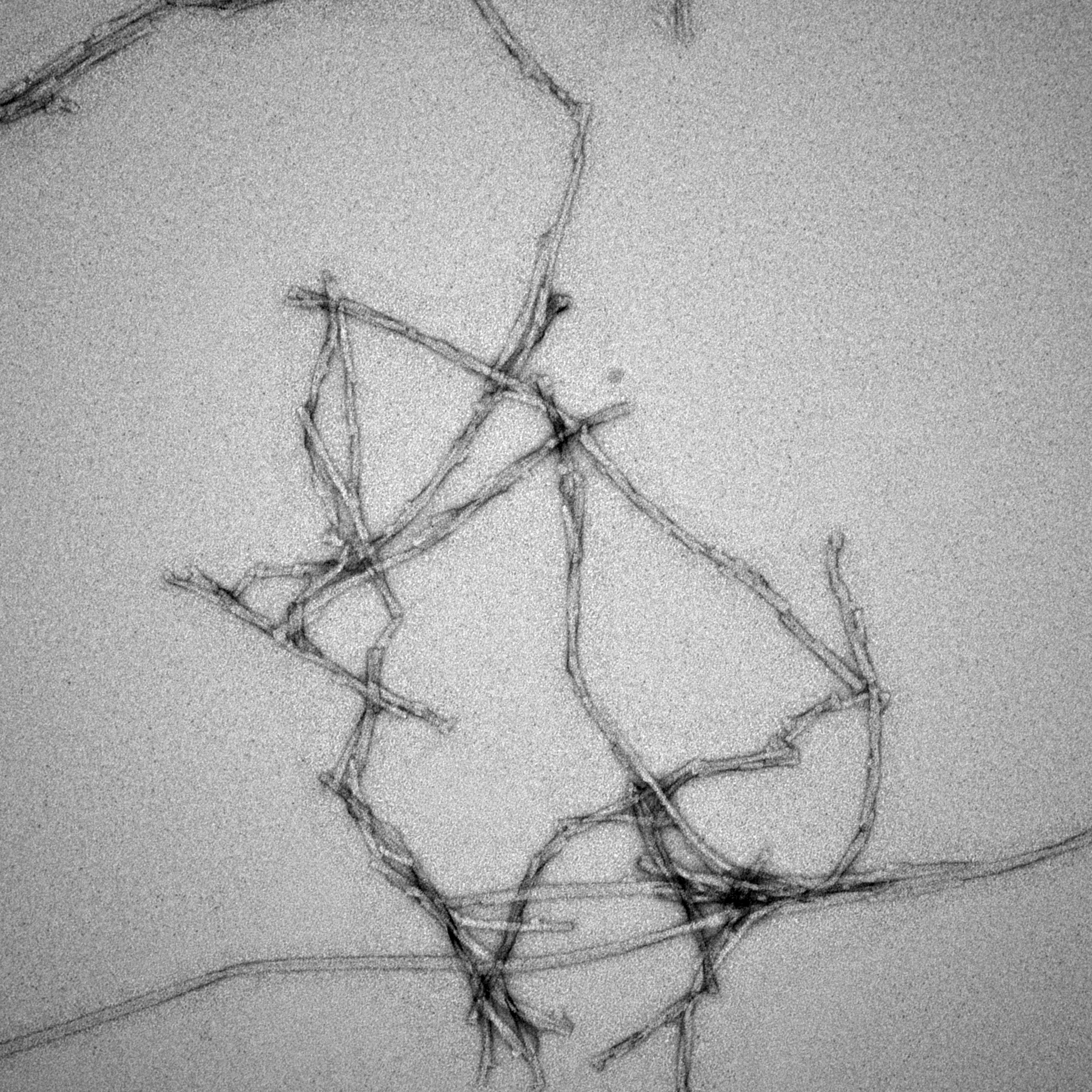

Supplement: Supplementary file 3 — Source data Fig. 1 [file 44318_2025_573_MOESM3_ESM.zip › Figure 1/1A/EM XG.png]

## Slide 1
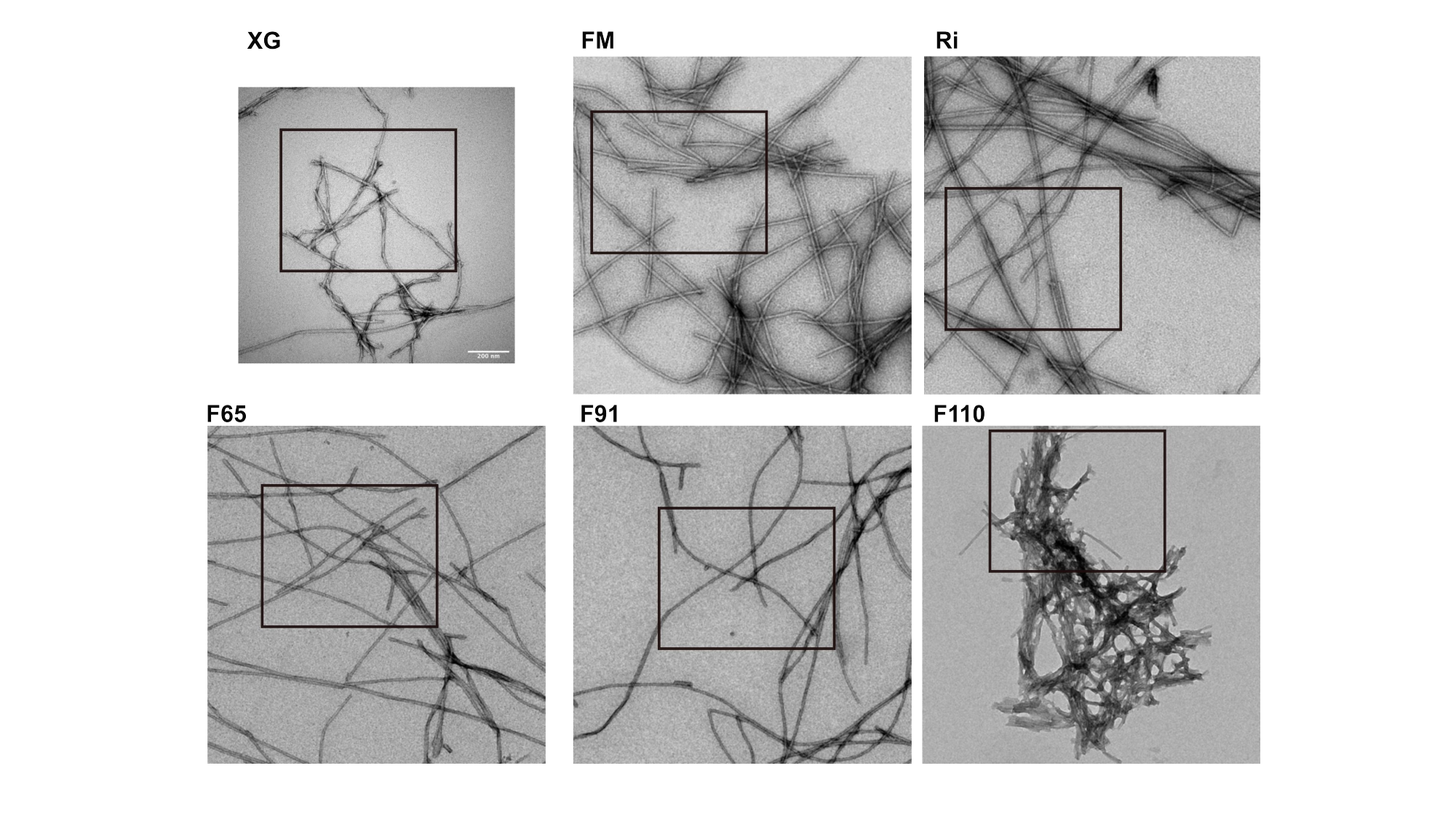

Supplement: Supplementary file 3 — Source data Fig. 1 [file 44318_2025_573_MOESM3_ESM.zip › Figure 1/1A/EM crop.pptx]

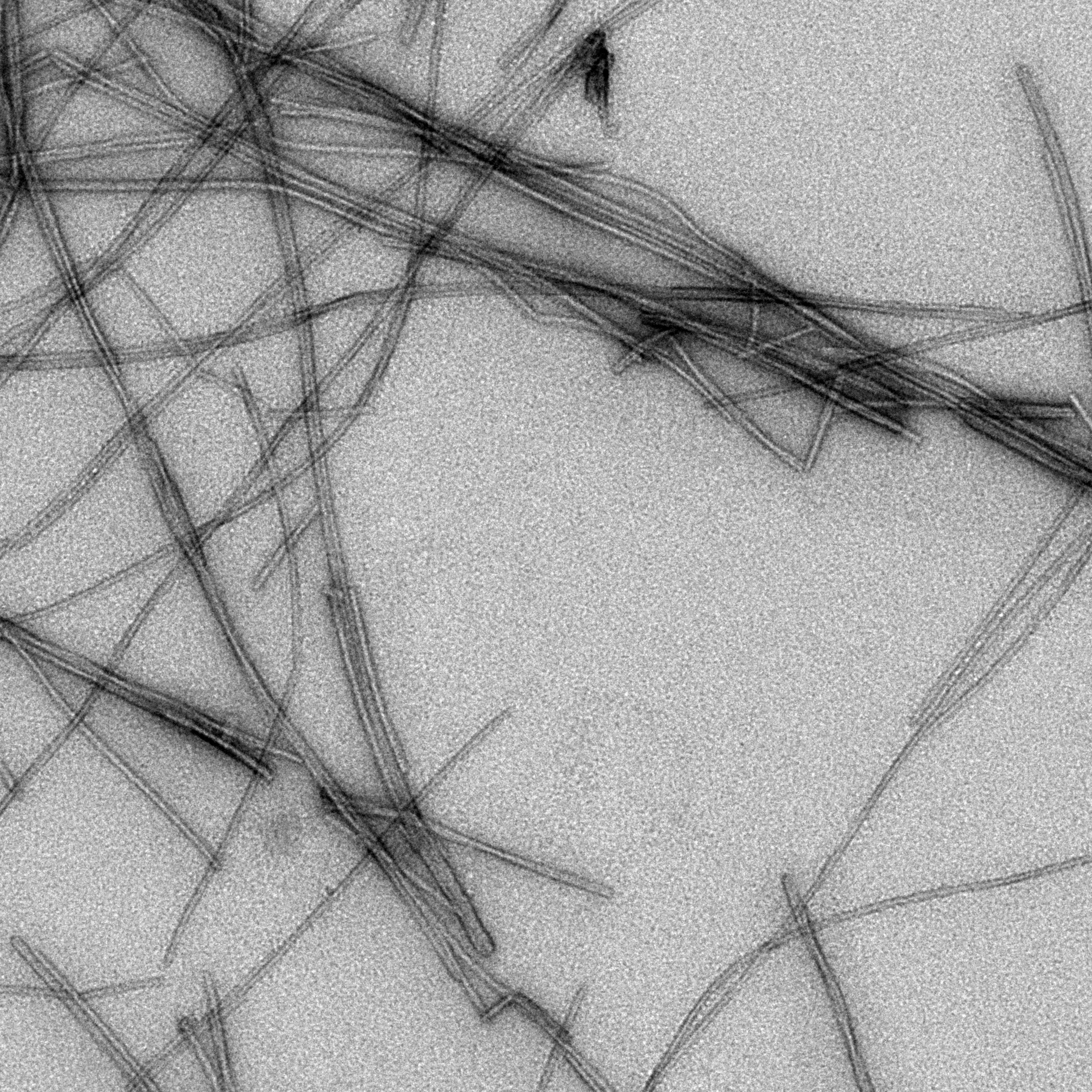

Supplement: Supplementary file 3 — Source data Fig. 1 [file 44318_2025_573_MOESM3_ESM.zip › Figure 1/1A/Ri_10kX_0005.jpg]

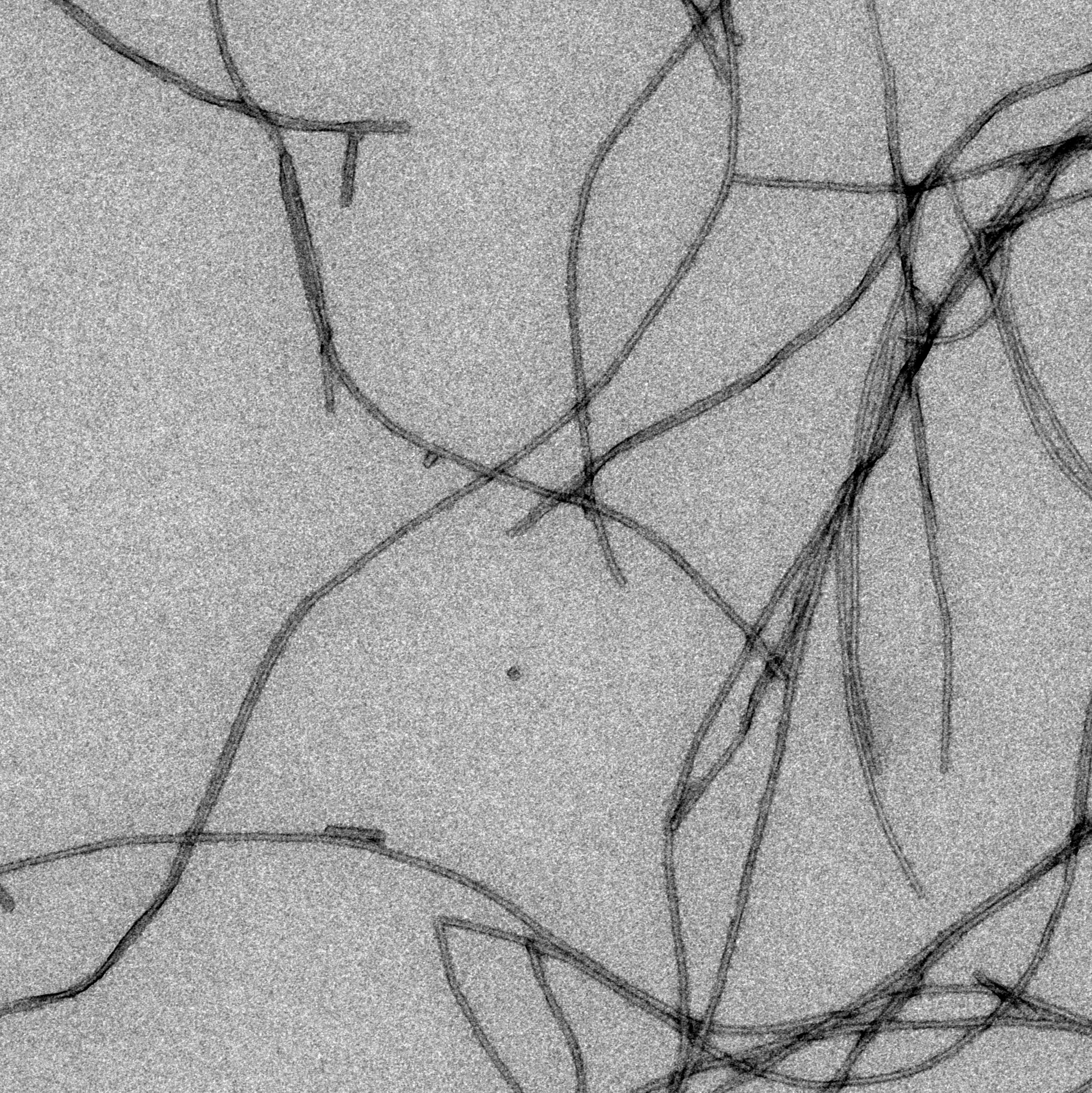

Supplement: Supplementary file 3 — Source data Fig. 1 [file 44318_2025_573_MOESM3_ESM.zip › Figure 1/1A/F91_10kX_0112.jpg]

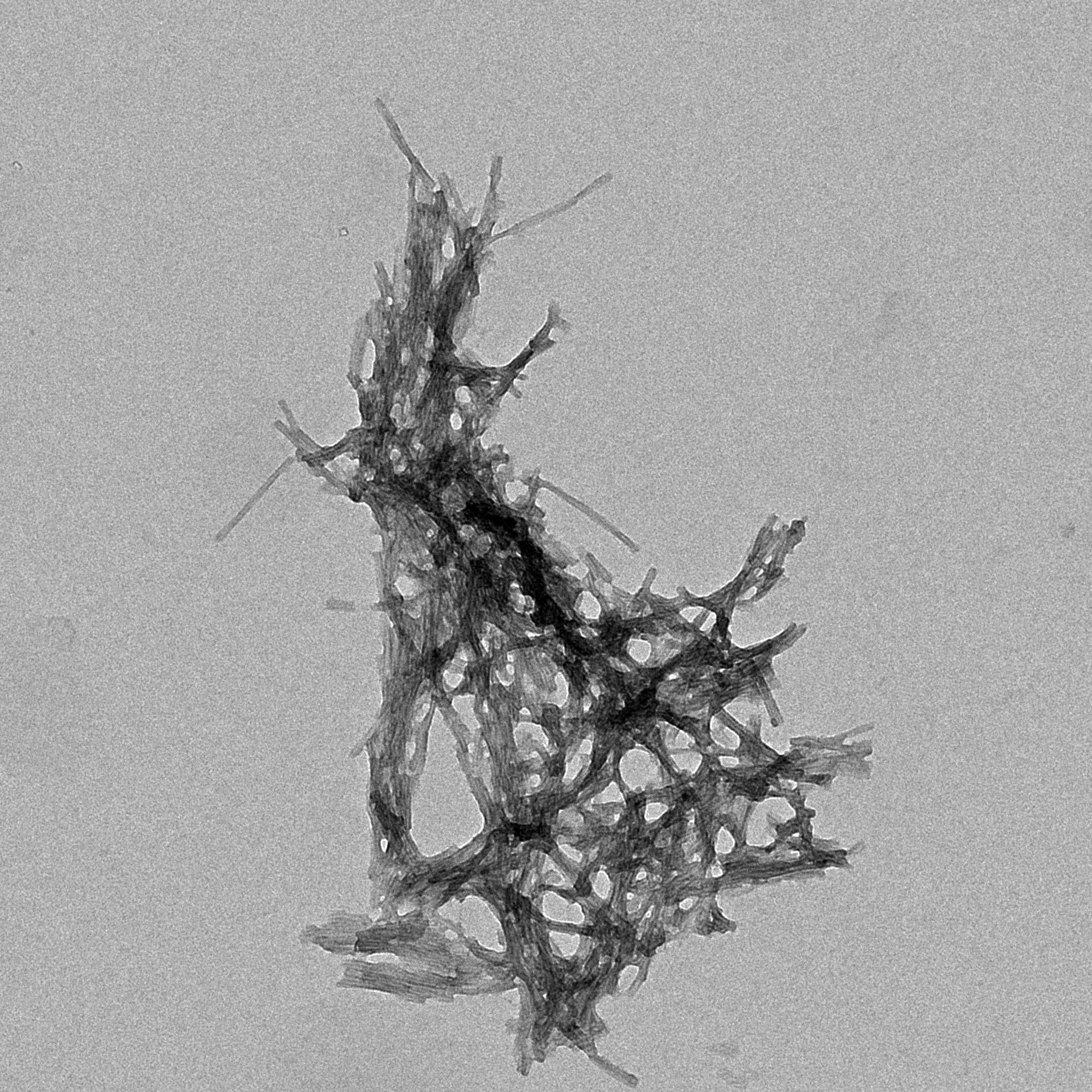

Supplement: Supplementary file 3 — Source data Fig. 1 [file 44318_2025_573_MOESM3_ESM.zip › Figure 1/1A/F110_10kX_0012.jpg]

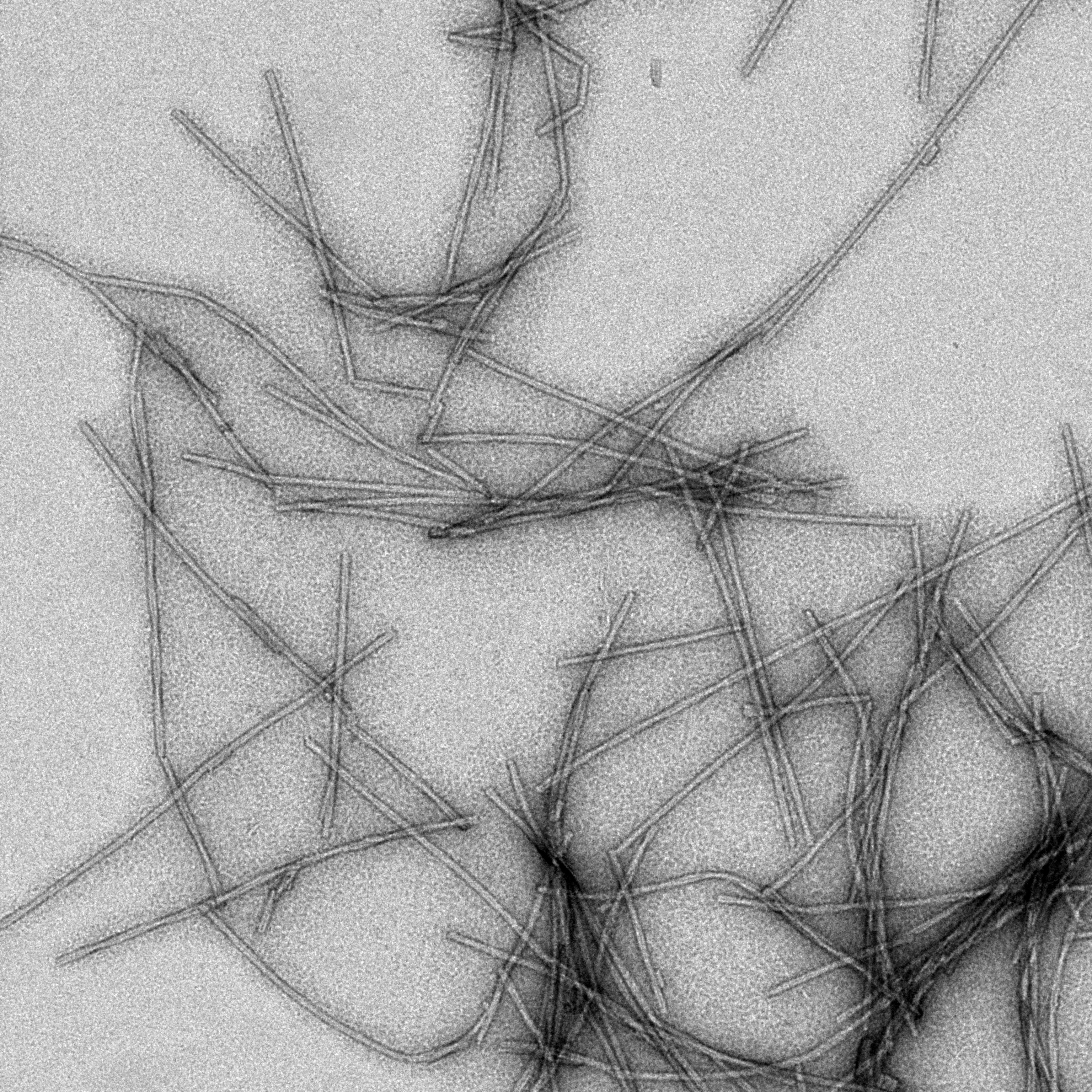

Supplement: Supplementary file 3 — Source data Fig. 1 [file 44318_2025_573_MOESM3_ESM.zip › Figure 1/1A/FM_10kX_0045.jpg]

Dataset shown in figure

XG - 3

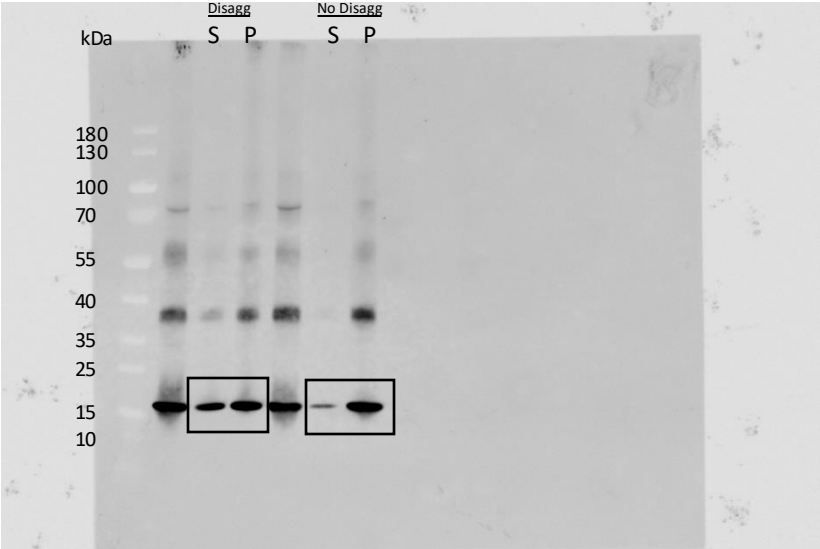

FM - 2

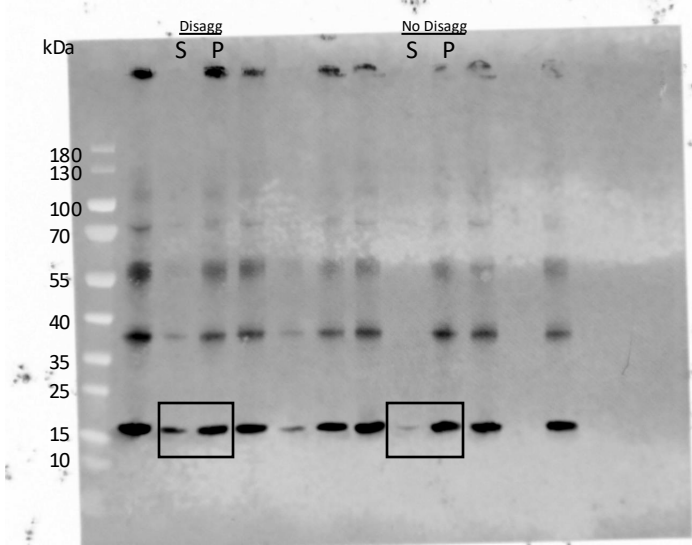

Ri -1

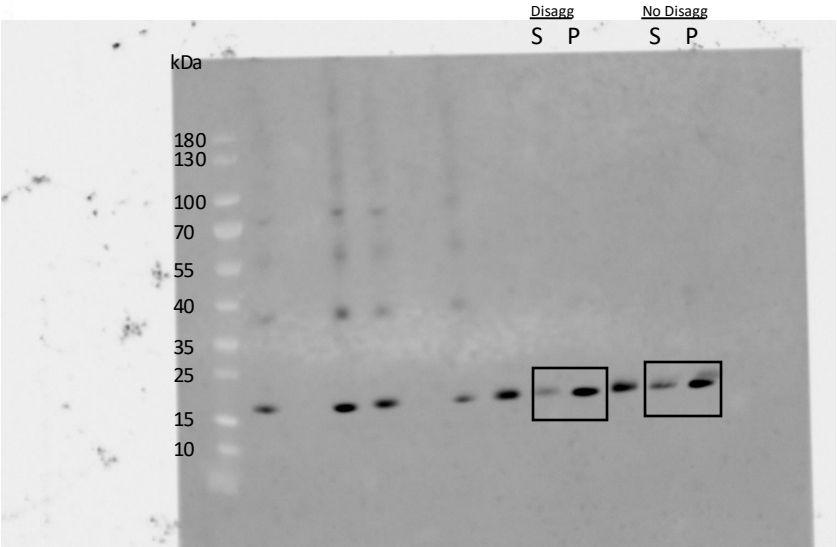

F65 - 2

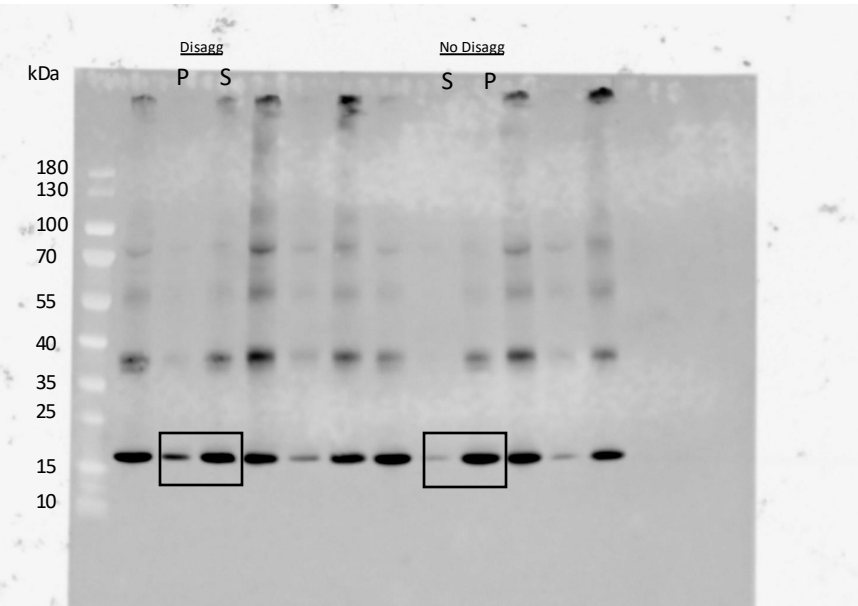

F91 - 1

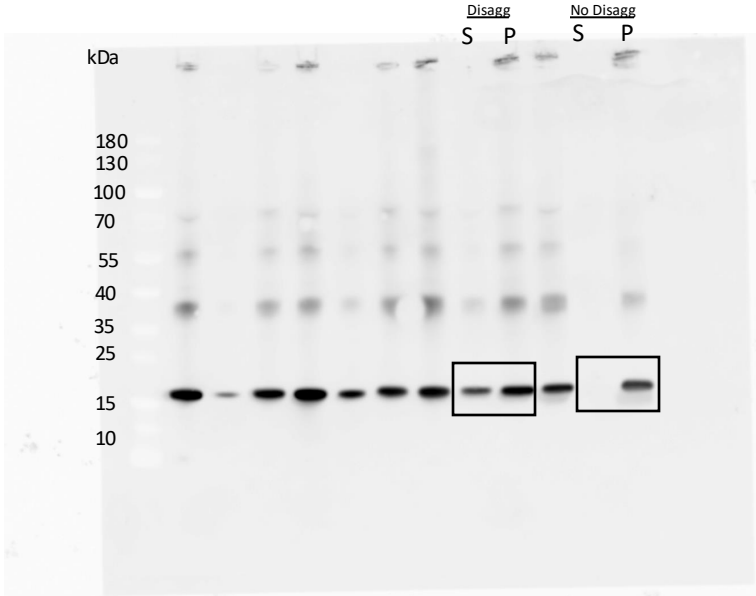

F110 - 1

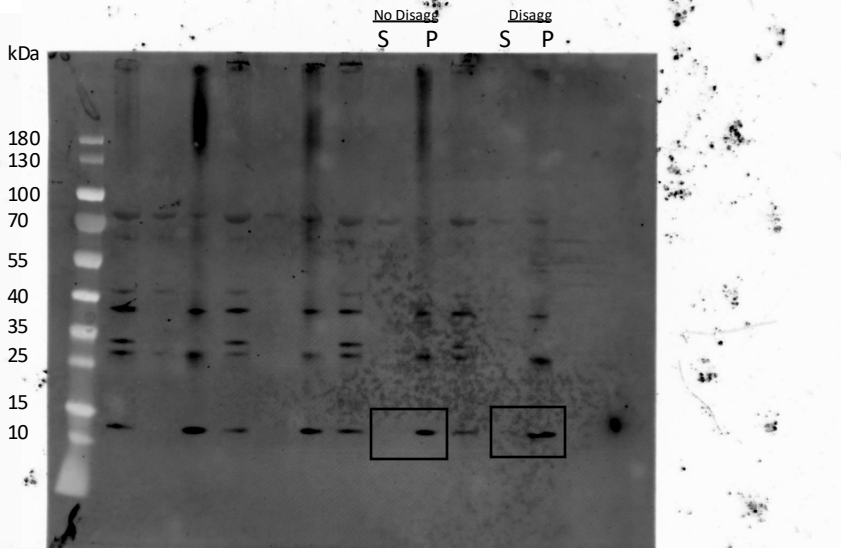

XG - 1

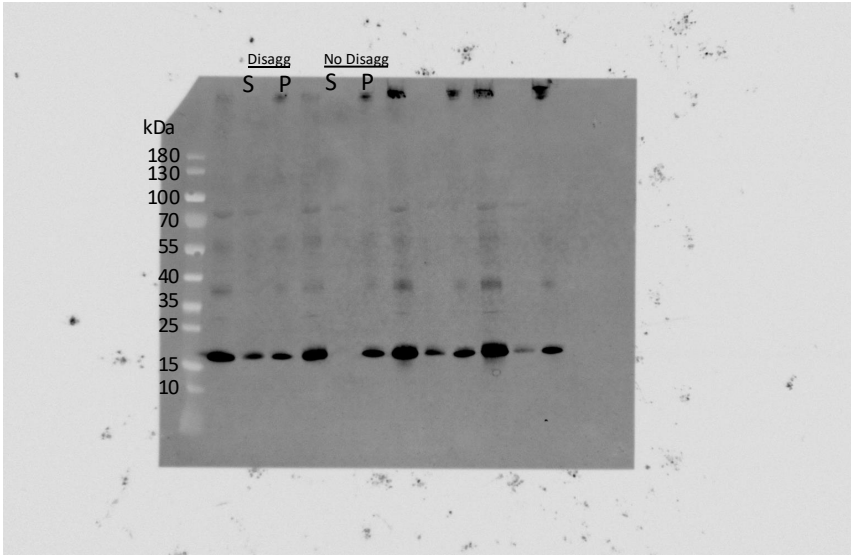

FM - 1

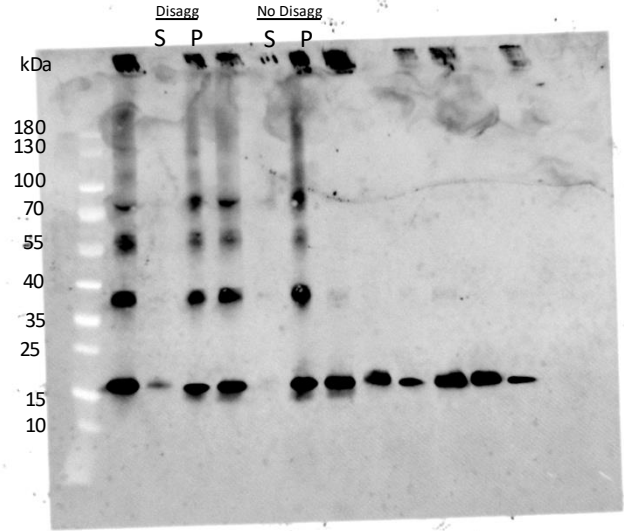

Ri - 2

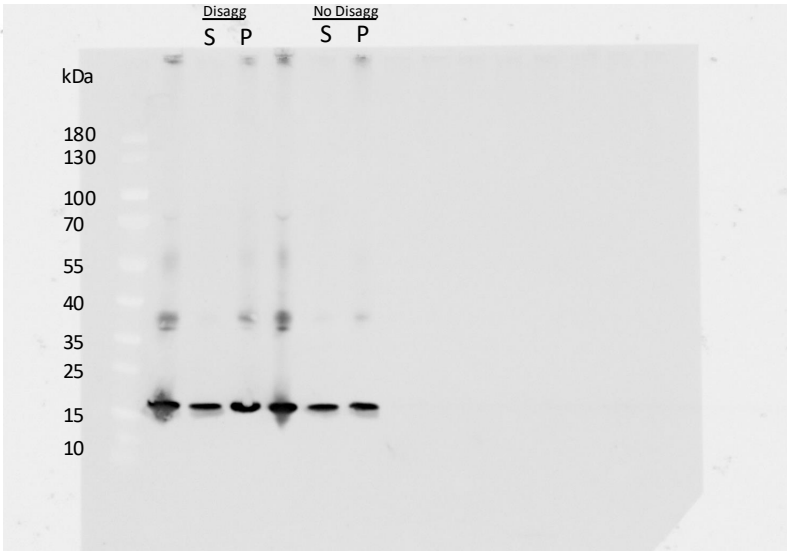

F65 - 1

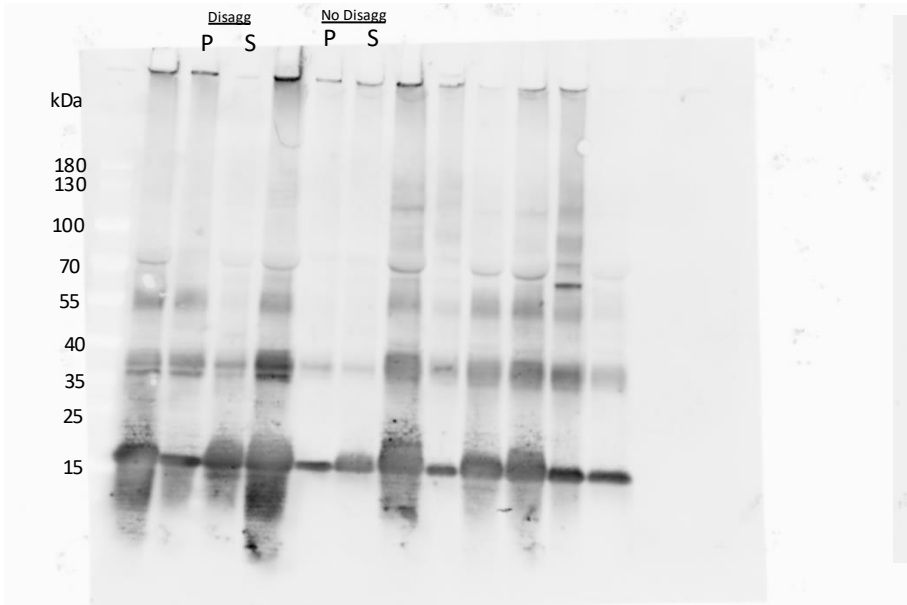

F91 - 2

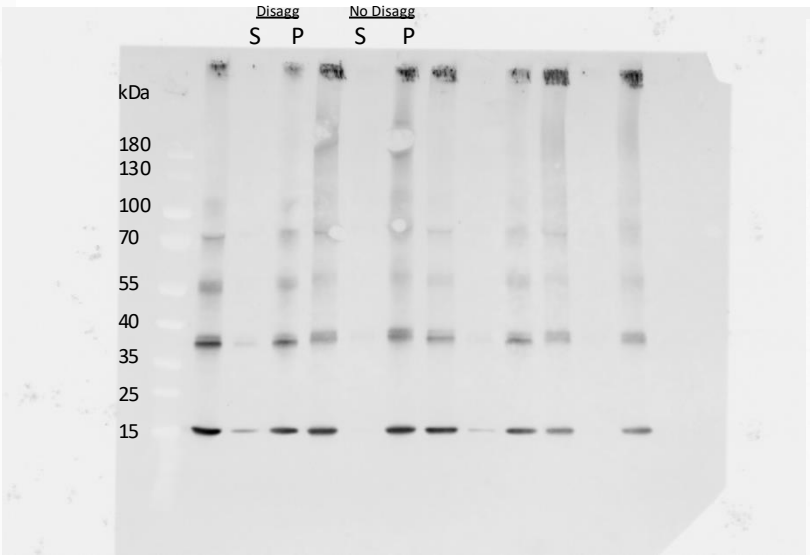

F110 - 2

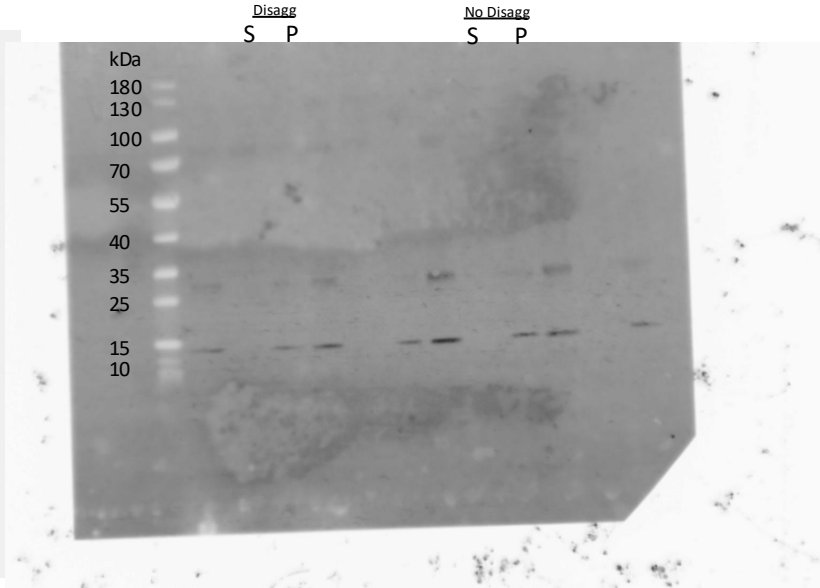

XG - 2

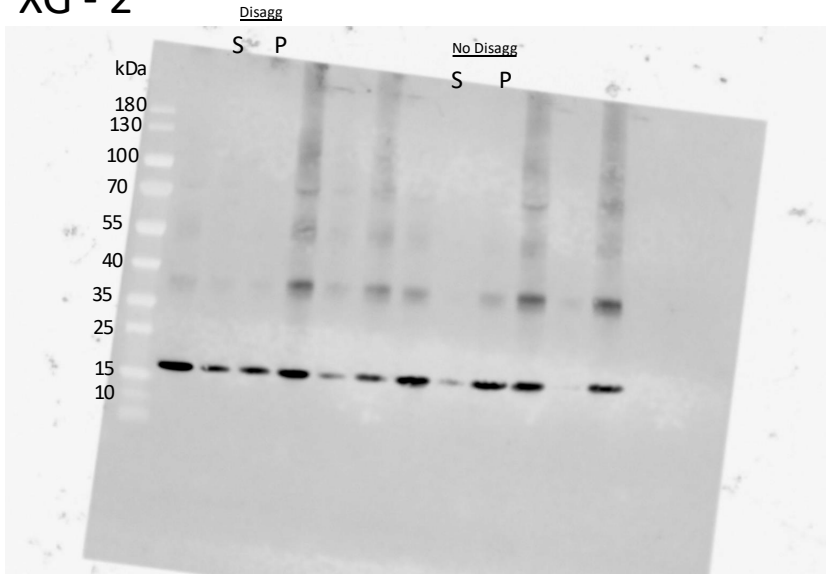

FM - 3

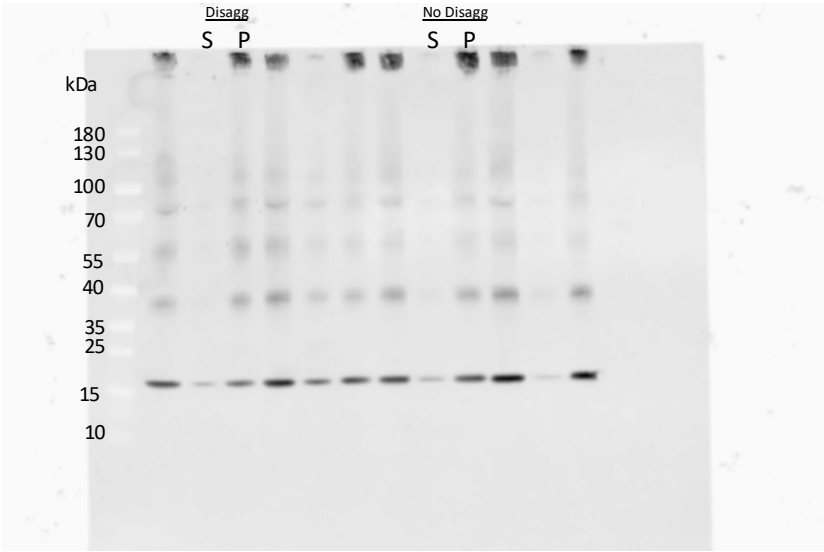

Ri - 3

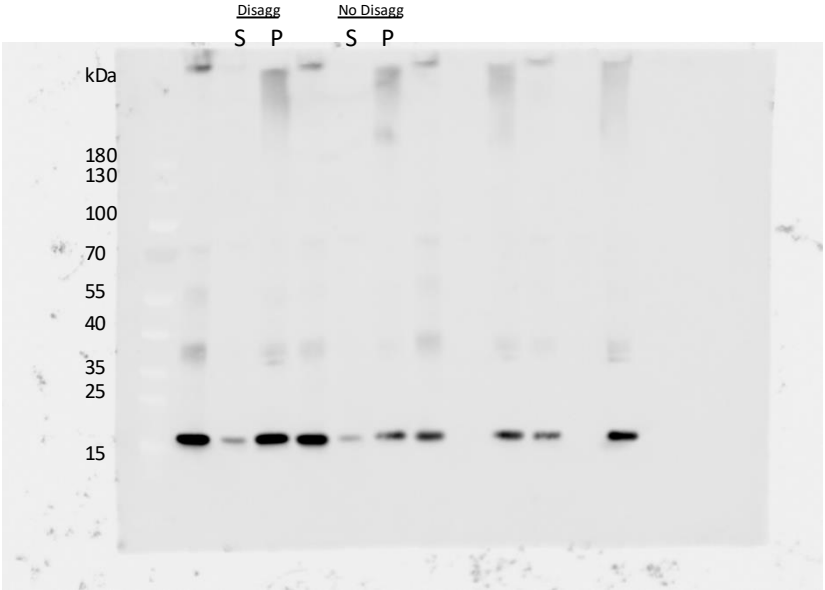

F65 - 3

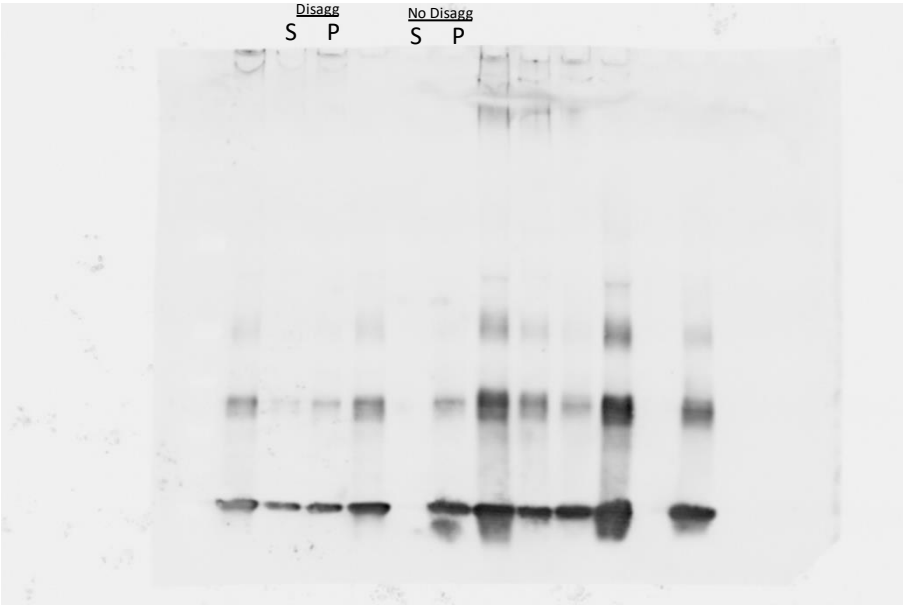

F91 - 3

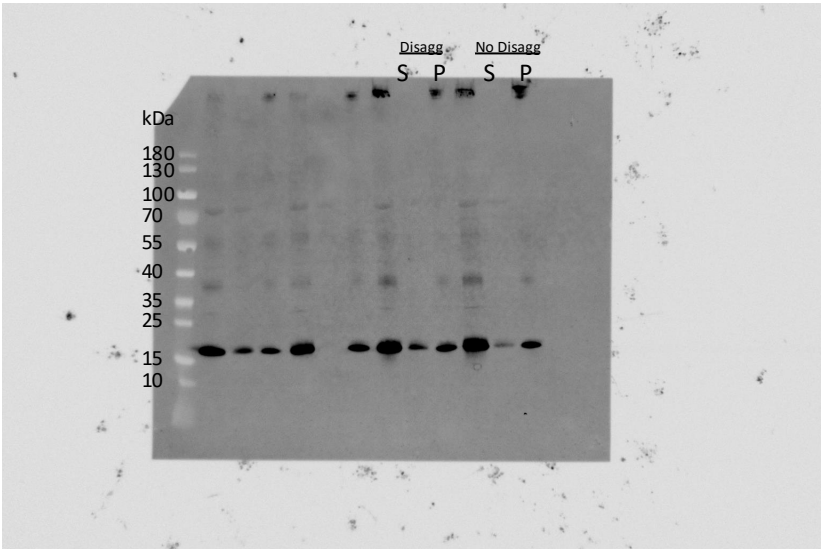

F110 - 3

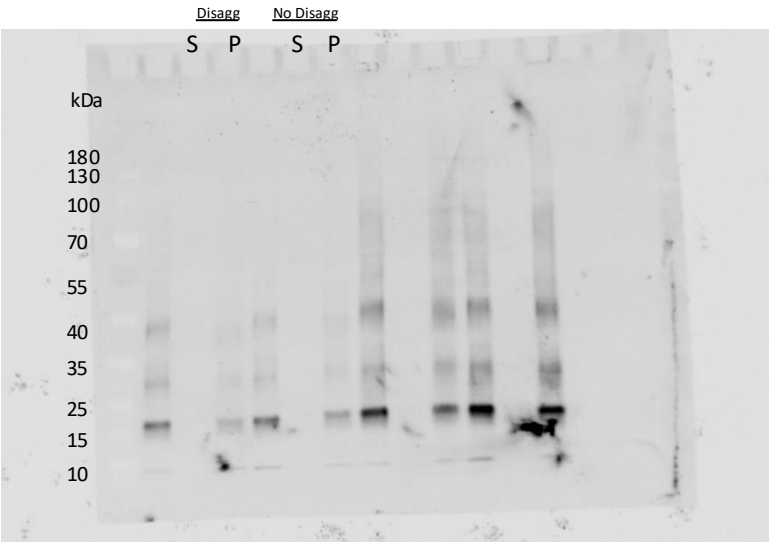

Supplement: Supplementary file 3 — Source data Fig. 1 [file 44318_2025_573_MOESM3_ESM.zip › Figure 1/1B/Uncropped western blots.pdf]

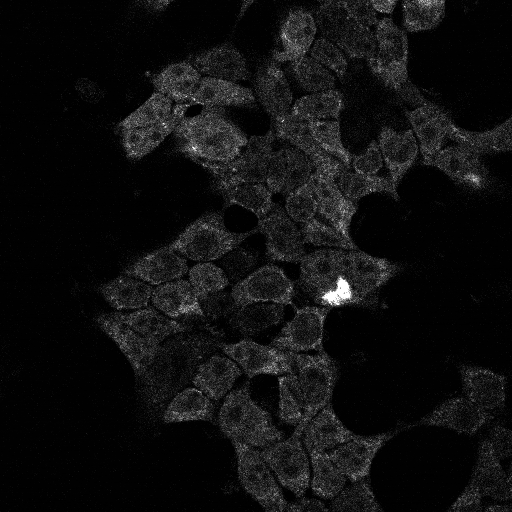

Supplement: Supplementary file 7 — Source data Fig. 5 [file 44318_2025_573_MOESM7_ESM.zip › Figure 5/5B/F65_no dissaggregation.tif]

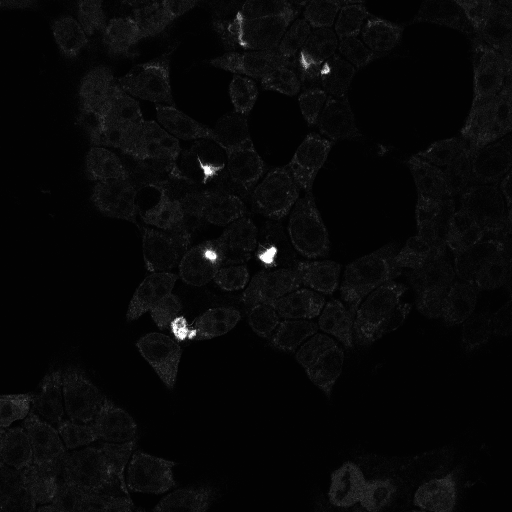

Supplement: Supplementary file 7 — Source data Fig. 5 [file 44318_2025_573_MOESM7_ESM.zip › Figure 5/5B/Fig5B_FM_Fibrils.tif]

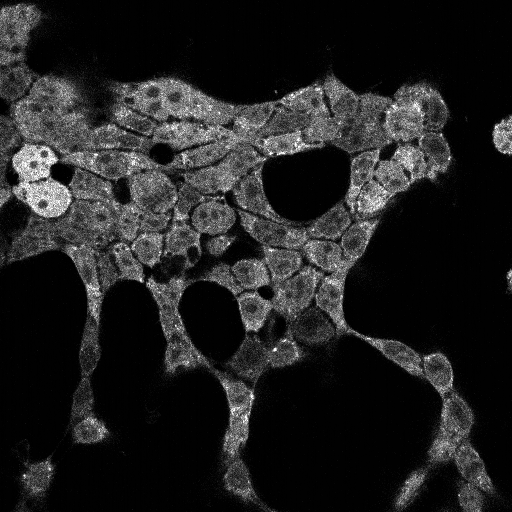

Supplement: Supplementary file 7 — Source data Fig. 5 [file 44318_2025_573_MOESM7_ESM.zip › Figure 5/5B/F65_monomers.tif]

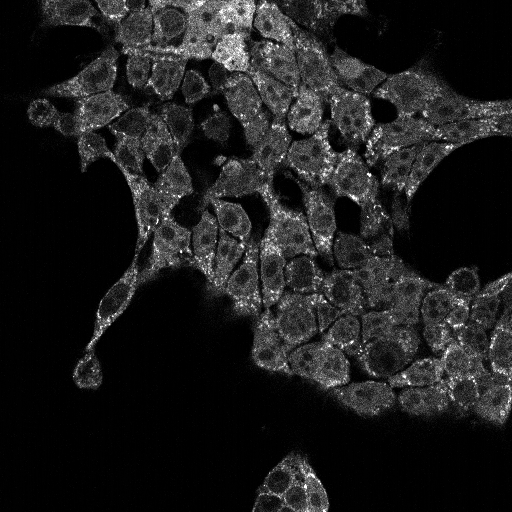

Supplement: Supplementary file 7 — Source data Fig. 5 [file 44318_2025_573_MOESM7_ESM.zip › Figure 5/5B/Fig5B_FM_Monomers.tif]

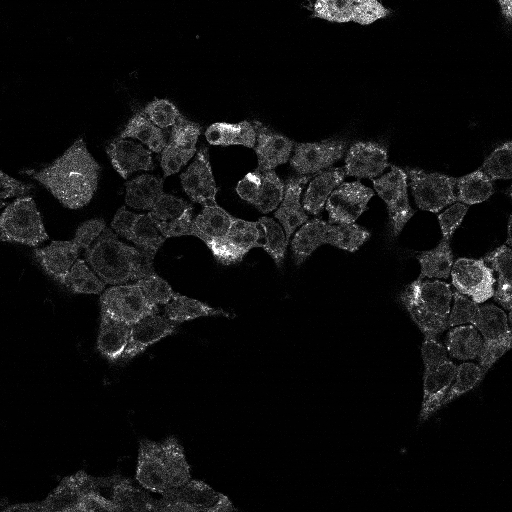

Supplement: Supplementary file 7 — Source data Fig. 5 [file 44318_2025_573_MOESM7_ESM.zip › Figure 5/5B/F65_disaggregation.tif]

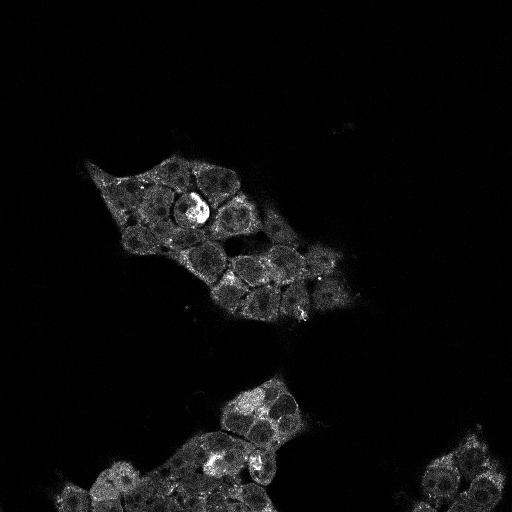

Supplement: Supplementary file 7 — Source data Fig. 5 [file 44318_2025_573_MOESM7_ESM.zip › Figure 5/5B/F65_Oligomers.tif]

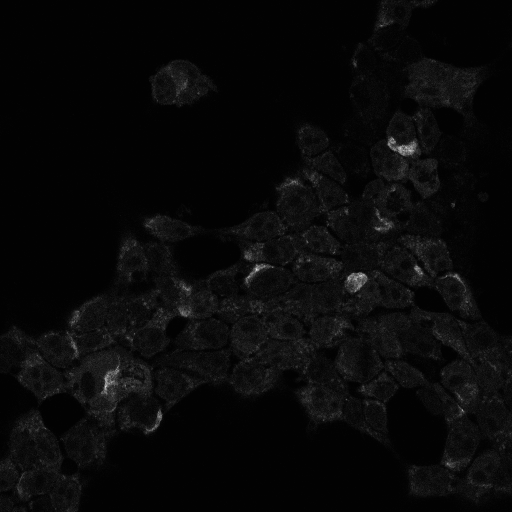

Supplement: Supplementary file 7 — Source data Fig. 5 [file 44318_2025_573_MOESM7_ESM.zip › Figure 5/5B/Fig5B_FM_smallFragments:Oligomers.tif]

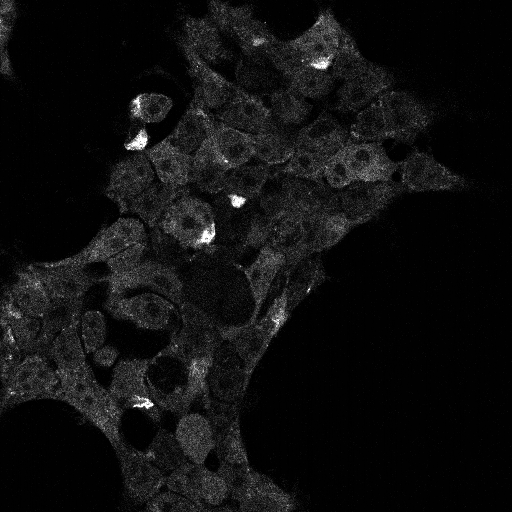

Supplement: Supplementary file 7 — Source data Fig. 5 [file 44318_2025_573_MOESM7_ESM.zip › Figure 5/5B/F65_fibrils.tif]

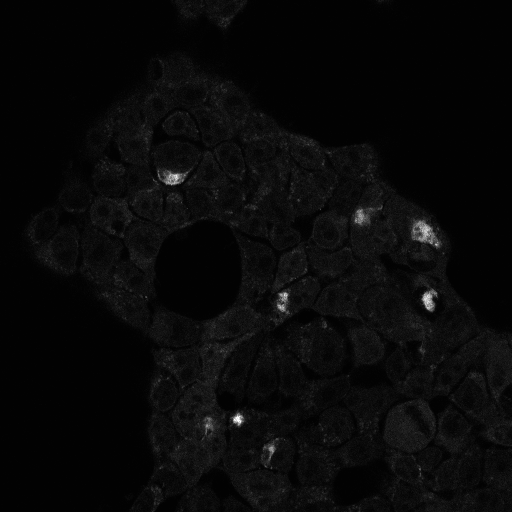

Supplement: Supplementary file 7 — Source data Fig. 5 [file 44318_2025_573_MOESM7_ESM.zip › Figure 5/5B/Fig5B_FM_Disaggregation.tif]

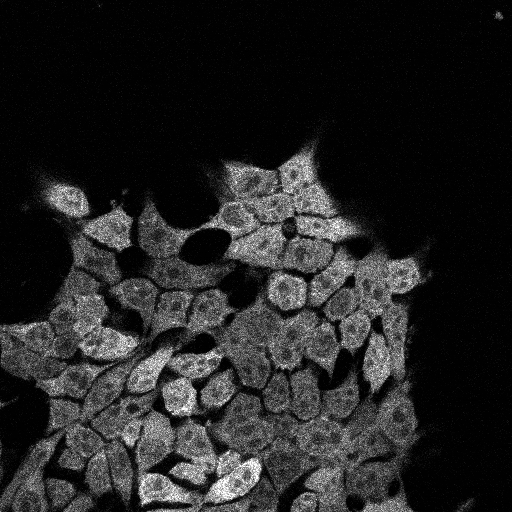

Supplement: Supplementary file 7 — Source data Fig. 5 [file 44318_2025_573_MOESM7_ESM.zip › Figure 5/5B/Fig5B_untreated.tif]

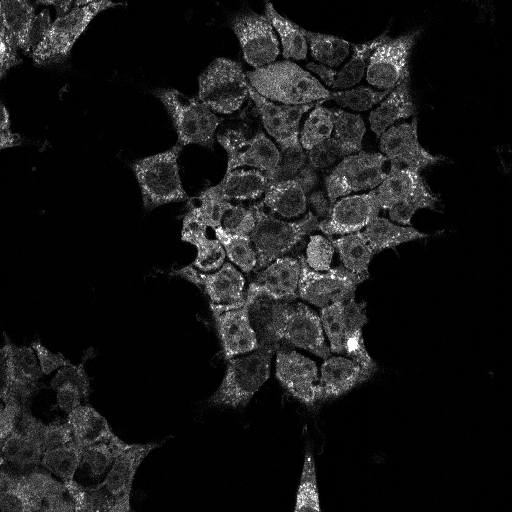

Supplement: Supplementary file 7 — Source data Fig. 5 [file 44318_2025_573_MOESM7_ESM.zip › Figure 5/5B/Fig5B_FM_NoDisaggregation.tif]
