# Supplementary material for: Structural polymorphism of α-synuclein fibrils alters the pathway of Hsc70-mediated disaggregation
Source: EMBO J. 2025 Oct 6;44(22):6499–526. doi: 10.1038/s44318-025-00573-3 (PMC12623964; doi:10.1038/s44318-025-00573-3)
Supplement: Supplementary file 8 — Expanded View Figures [file 44318_2025_573_MOESM8_ESM.pdf]

Expanded View Figures

Figure EV1. High chemical stability of ribbons and F110 polymorphs could explain their resistance to chaperone-mediated disaggregation.

(**A**) SDS-PAGE of α-syn monomers released to supernatant, separated from fibrillar material by centrifugation, after incubation of fibrils on ice for the indicated times. (**B**) Fraction of total fibrils depolymerized, as shown in (**A**), as a function of incubation time on ice. (**C**) Correlation plot of disaggregation after 16 h in percent (mean ± s.e.m.) and fraction depolymerized with a linear correlation fit (solid line ($r = 0.72$)). (**D**) ThT disaggregation assay with 1× (XG, blue; F91, red; F65, green; FM, yellow; Ri, gray; F110, purple) or 2× (black) chaperone concentration. Representative graphs of three technical replicates are shown.

 © The Author(s)

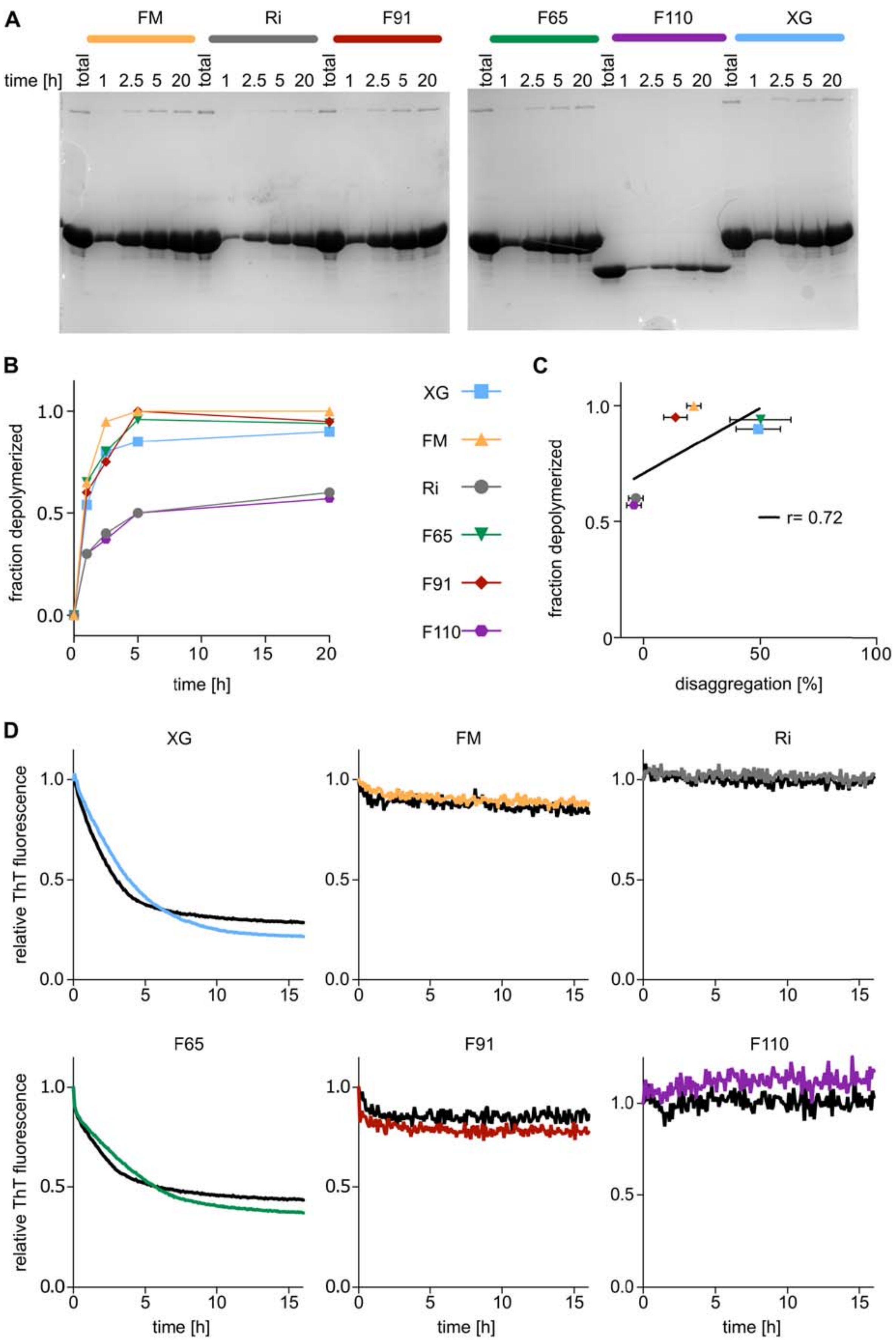
A
FM
Ri
F91
F65
F110
XG
time [h]
total 1 2.5 5 20
B
fraction depolymerized
time [h]
XG
FM
Ri
F65
F91
F110
C
fraction depolymerized
disaggregation [%]
r= 0.72
D
XG
FM
Ri
F65
F91
F110
relative ThT fluorescence
time [h]

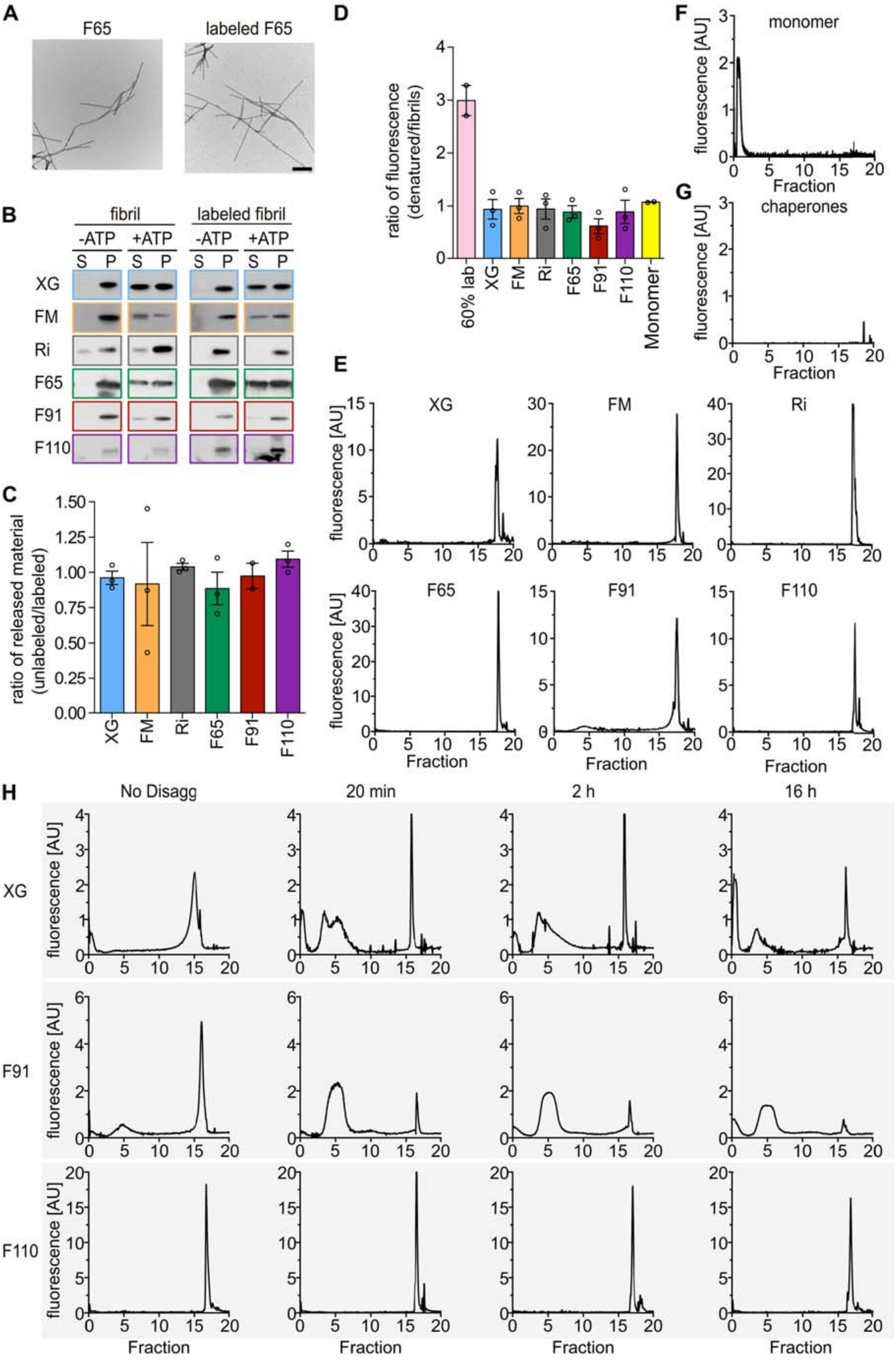
A
F65
labeled F65
B
fibril
labeled fibril
-ATP
+ATP
S P S P S P S P
XG
FM
Ri
F65
F91
F110
C
ratio of released material (unlabeled/labeled)
XG FM Ri F65 F91 F110
D
ratio of fluorescence (denatured/fibrils)
60% lab XG FM Ri F65 F91 F110 Monomer
E
XG FM Ri F65 F91 F110
fluorescence [AU]
Fraction
F
monomer
G
chaperones
H
No Disagg
20 min
2 h
16 h
XG
F91
F110

 © The Author(s)

Figure EV2. Fibrillar fragments accumulate to various degrees during chaperone-mediated disaggregation of polymorphs.

(**A**) Negative stain EM images of polymorphs F65 unlabeled (left) and AF555-labeled (right) (scale bar 200 nm). (**B**) Representative western blot images of unlabeled (left) and AF555-labeled polymorphs (right) incubated with the chaperone machinery in the presence (+ATP) and absence of ATP (−ATP). (**C**) Ratio of released α-syn protein of AF555-labeled and unlabeled fibrils of all polymorphs. Quantification of protein in the supernatant of the total (P + S) after 16 h disaggregation by the active chaperone machinery in (**B**) (analyzed with ImageJ). Data are mean ± s.e.m., $n = 3$, except polymorph F91 where $n = 2$. (**D**) Ratio of normalized fluorescence of denatured AF555-labeled fibrils/monomer in GnHCl compared to labeled fibrils/monomer in buffer. Data are mean ± s.e.m., $n = 3$, except 60% labeled fibrils and monomer where $n = 2$. (**E**, **F**) Sucrose density gradient (10–85%) profile of AF555-labeled monomers (**E**) and chaperones (Hsc70, DnaJB1, Apg2) only (**F**). Sucrose gradient of labeled fibrils (XG, FM, Ri, F65, F91, F110) is shown in (**G**). (**H**) Representative sucrose gradient (10–85%) profile of a polymorph XG, F65, F91, and F110 after incubation of AF555-labeled fibrils with the active chaperone machinery (Hsc70, DnaJB1, Apg2, + ATP) for 20 min, 2 h, 16 h, and 16 h incubation with the inactive machinery (−ATP, No Disaggregation).

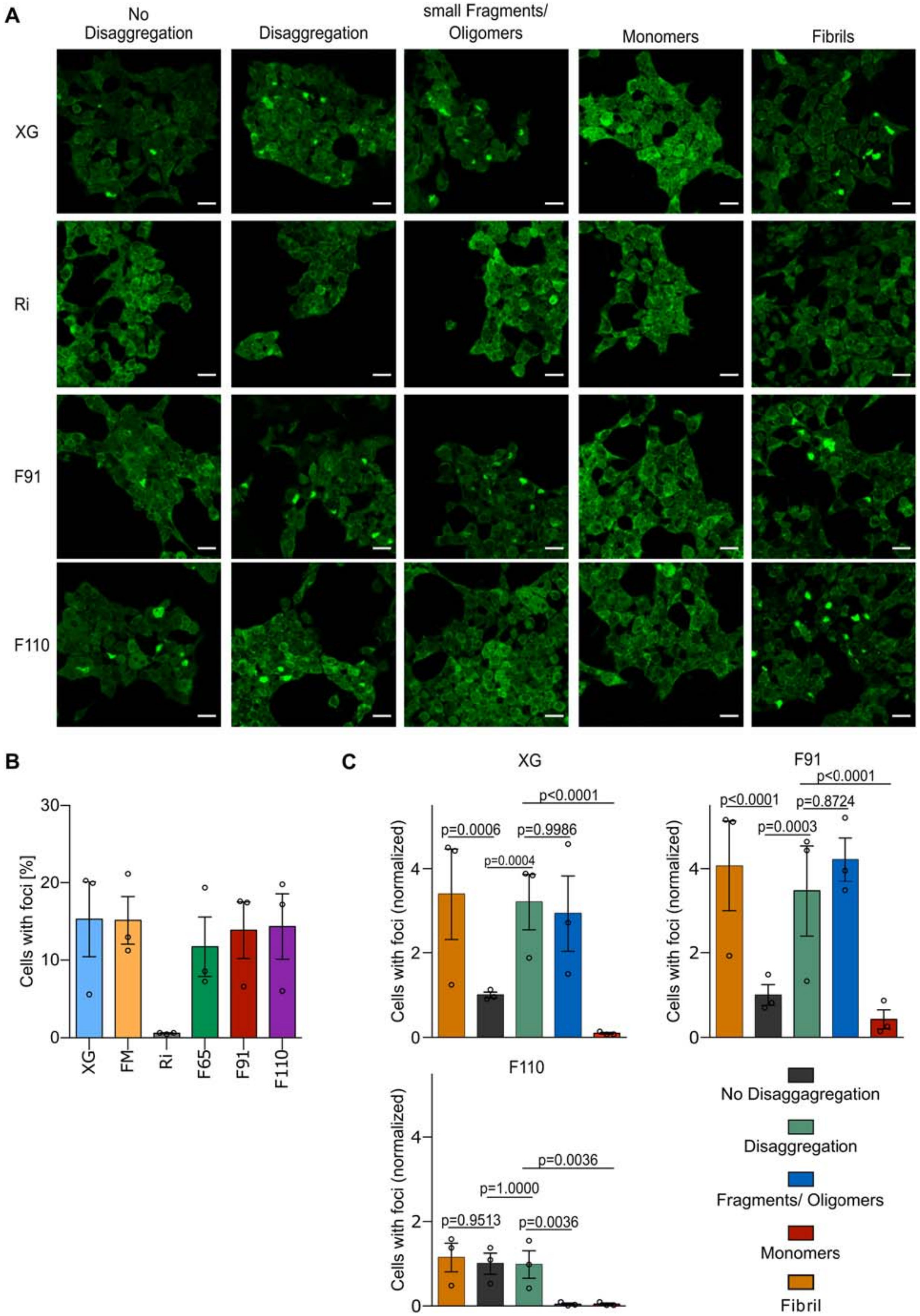
A
No Disaggregation
Disaggregation
small Fragments/ Oligomers
Monomers
Fibrils
XG
Ri
F91
F110
B
Cells with foci [%]
XG
FM
Ri
F65
F91
F110
C
XG
F91
F110
Cells with foci (normalized)
p=0.0006
p=0.0004
p=0.9986
p<0.0001
p<0.0001
p=0.0003
p=0.8724
p=0.9513
p=1.0000
p=0.0036
No Disaggregation
Disaggregation
Fragments/ Oligomers
Monomers
Fibril

 © The Author(s)

Figure EV3. Disaggregation reaction products trigger foci formation in the human cell model.

(**A**) Representative fluorescence intensity microscopic images of HEK293T cells stably expressing *α*-syn A53T-YFP seeded and treated with fibril preparations of polymorph XG, Ri, F91, and F110. The cells were exposed to fibrils only, fibrils incubated with chaperones in the absence (No Disaggregation) and presence of ATP (Disaggregation), as well as small fragments/oligomer and monomer fractions of the disaggregation reaction separated by centrifugation as described in Fig. 4C. Scale bar corresponds to 20 µm. (**B**) Percentage of cells with foci for the individual polymorphs ($n = 3$). (**C**) Quantification of the percentage of cells with foci normalized to the No disaggregation sample (No disaggregation, black; Disaggregation, green; small fragments/oligomers, blue; monomers, red; fibrils, orange). The Ri polymorph did not induce aggregation in the used reporter cell system (in (**B**)) and was therefore not further analyzed. Data are mean ± s.e.m., $n = 3$. Statistical analysis was performed by nonparametric two-way ANOVA with pairwise comparisons of estimated marginal means with Tukey correction for multiple comparisons. Exact p-values are indicated in the figure.

© The Author(s)

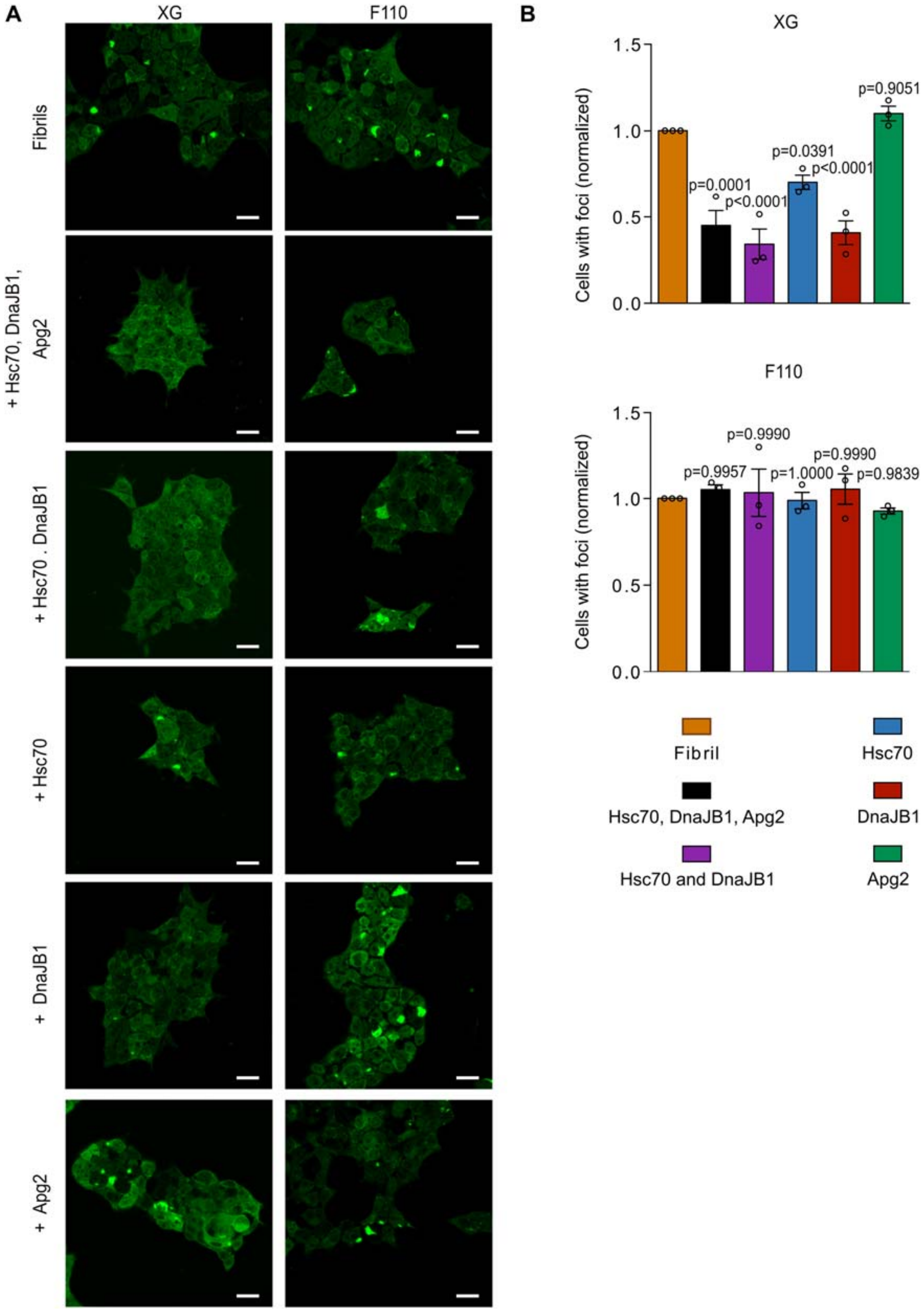
A
XG
F110
Fibrils
+ Hsc70, DnaJB1, Apg2
+ Hsc70 . DnaJB1
+ Hsc70
+ DnaJB1
+ Apg2
B
XG
Cells with foci (normalized)
1.5
1.0
0.5
0.0
p=0.0001
p<0.0001
p=0.0391
p<0.0001
p=0.9051
F110
Cells with foci (normalized)
p=0.9957
p=0.9990
p=1.0000
p=0.9990
p=0.9839
Fibril
Hsc70, DnaJB1, Apg2
Hsc70 and DnaJB1
Hsc70
DnaJB1
Apg2

Figure EV4. Pre-incubation of α-syn fibrils with DNAJB1 reduces foci formation.

(**A**) Representative fluorescence intensity microscopic images of HEK293T cells stably expressing α-syn A53T-YFP seeded with fibrils (polymorphs XG and F110) incubated with different chaperone combinations; fibrils without chaperones as a control, fibrils with the whole chaperone machinery (Hsc70, DnaJB1, Apg2), Hsc70 and DnaJB1 and Hsc70, DnaJB1 and Apg2 individually. Scale bar corresponds to 20 µm. (**B**) Quantification of the percentage of cells with foci normalized to fibril-only conditions (XG, top; F110, bottom). Data are mean ± s.e.m., $n = 3$. Statistical analysis was performed by nonparametric two-way ANOVA with pairwise comparisons of estimated marginal means with Tukey correction for multiple comparisons. Exact p-values are indicated in the figure.

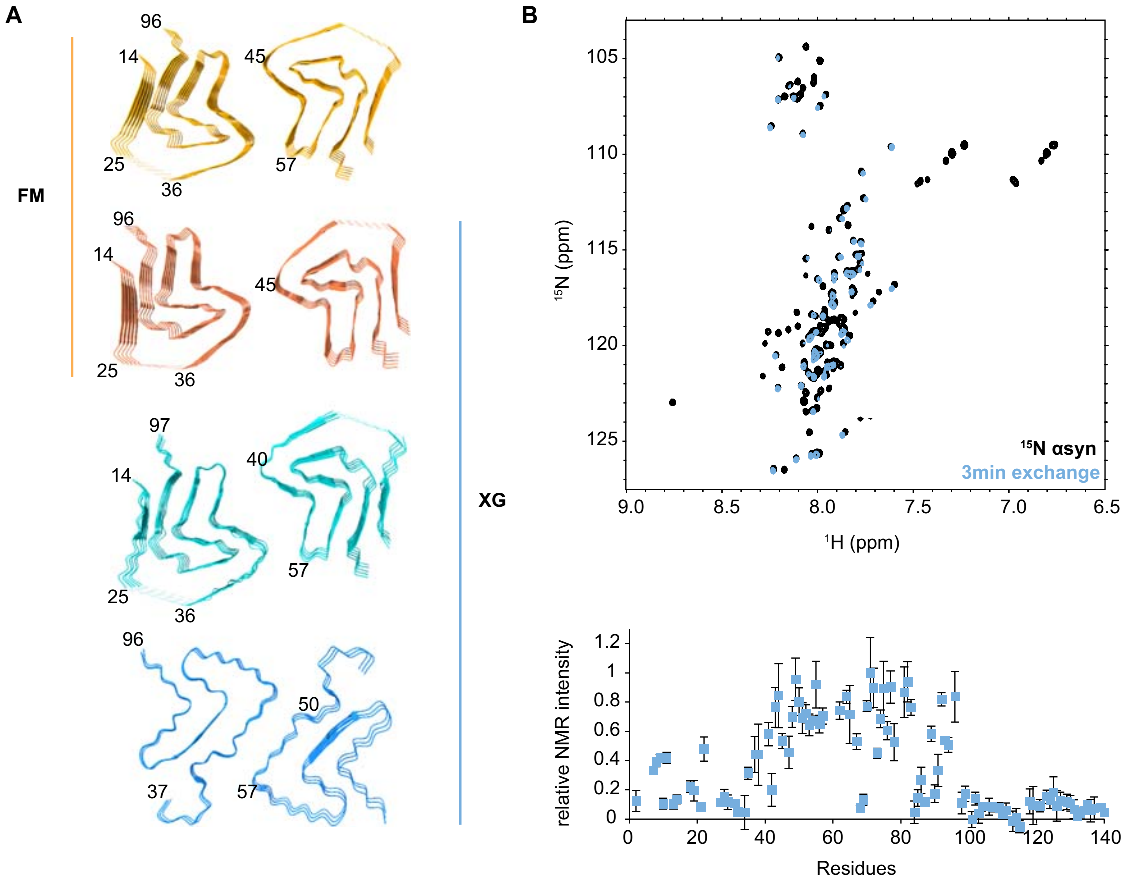

Figure EV5. Structural comparison of α-syn polymorphs FM and XG.

(**A**) Cryo-electron microscopy-derived structural models of different fibril conformations generated under the FM (PDB: 6SSX and 6SST) (Guerrero-Ferreira et al, 2019) and XG (PDB: 8RRR and 8RQM) (Monistrol et al, 2025) aggregation conditions. (**B**) Hydrogen–deuterium exchange of the XG polymorph followed by NMR spectroscopy. NMR resonance intensities after 3 min of HD exchange in deuterated buffer are plotted relative to their non-exchange intensities as a function of the α-syn sequence. The N- and C-terminal sequences exchange rapidly and are thus mostly disordered, while residues 38–96 make up the slow-exchanging fibril core. Data are mean ± s.d., $n = 3$.

 © The Author(s)
